# Analysis of instrumentation failures after three column osteotomies of the spine

**DOI:** 10.1186/s13013-017-0127-x

**Published:** 2017-06-05

**Authors:** Niranjan Kavadi, Richard A. Tallarico, William F. Lavelle

**Affiliations:** 1 Department of Orthopedic Surgery, SUNY Upstate Medical University, 750 E. Adams Street, Syracuse, NY 13210 USA; 2 6620 Fly Road, Suite 200, East Syracuse, NY 13057 USA

**Keywords:** Spinal deformity, Fixed sagittal imbalance, Tricolumnar osteotomy, Pseudoarthrosis, Implant rod failure, Lumbosacral junction

## Abstract

**Background:**

Correction of fixed spinal imbalance in a sagittal and/or coronal plane frequently needs a tricolumnar wedge resection when the deformity is rigid. Complications associated with deformity correction surgery are pseudoarthrosis and implant failure located along the construct. The purposes of this study were to assess comparative rates of pseudoarthrosis (implant failure) at weaker points along lumbosacral junction and level of osteotomy, estimate overall incidence of implant failure, and comparatively analyze failures at different points along the construct.

**Methods:**

This was an IRB approved, single center study retrospective analysis. Twenty-six patients who underwent three column osteotomies were grouped according to procedure: pedicle subtraction osteotomy (PSO, (*n* = 18)); vertebral column resection (VCR, (*n* = 4)); hemivertebra excision (HE, (*n* = 2)); and extracavitary corpectomy (EC, (*n* = 2)). Follow-up data is presented on all of the study patients. Number of levels of fusion, anchors, percent saturation of fixation levels, type of bone graft and graft substitutes, and rod material and diameter were recorded. Radiographical data was reviewed preoperatively and postoperatively at 2 weeks and 3, 6, and 12 months and annually to determine sagittal and coronal balance, lumbopelvic parameters, presence or absence of interbody structural support, laterality or rod failure, and time to implant failure.

**Results:**

Twenty-seven percent (7/26) patients demonstrated rod breakage either unilaterally (*N* = 2) or bilaterally (*N* = 5) during follow-up. Seventy-one percent had increasing back pain or worsening sagittal balance, while remaining failures found incidentally. No failures in children were seen.

**Conclusion:**

Tricolumnar osteotomy by posterior approach is a valuable tool. Rod failures found approximately 1 year from surgery, with 86% located at level of osteotomy and 14% at lumbosacral junction. Possible reasons are increased stress in the rod at this point and relatively deficient bone stock secondary to wide laminectomy. The low rate of rod breakage at lumbosacral junction may be related to adoption of structural interbody graft and stronger iliac screws. Additional biomechanical studies needed to assess the importance of these factors. This was a level IV study.

## Background

Fixed sagittal imbalance can be defined as a syndrome in which the patient is unable to stand erect without flexing knees and hips [1]. With advancing age, progressive degenerative changes in the disc lead to gradual loss of lumbar lordosis. Over time distal lumbar segments and the lumbosacral junction fail to compensate for positive sagittal balance. Pelvic alignment changes with worsening posture finally lead to fixed changes in spinopelvic parameters: lumbar lordosis, pelvic tilt, sacral slope, and sagittal vertical axis. Fixed sagittal imbalance with alteration in these spinopelvic parameters relates to increased energy expenditure resulting in declining quality of life [2, 3]. Hence, correction of these parameters while treating sagittal imbalance is critical.

Optimal sagittal balance can be achieved surgically with utilization of different types of osteotomies. Two broad groups are as follows: osteotomies involving only the posterior column of the spine and tricolumnar osteotomies achieving correction through all three columns of the spine. The Smith-Petersen [4–6] osteotomy involves resection of only posterior elements in a spinal segment with an intact mobile disc leading to shortening of posterior column and lengthening of the anterior column of the spine. Pedicle subtraction osteotomy (PSO) [7–10] entails removal of a tricolumnar wedge when more significant correction is required. Shortening of the posterior column is achieved without lengthening the anterior column with no risk to the anterior soft tissues. PSO outcomes to correct fixed sagittal malalignment have been well documented in literature [11]. Vertebral column resection (VCR) [12–15] involves complete resection of one or more vertebral segments and is applied for correction of moderate to severe spinal deformities, including large rigid curves and fixed trunk translation.

Successful outcomes of deformity correction require maintenance of the optimal sagittal and coronal balance over time. High rates of pseudoarthrosis have been reported secondary, due to a large number of segments fused [16, 17]. Implant failures are commonly located at weak points along the construct, the lumbosacral junction, and the area of previous laminectomies. Different strategies have utilized L5–S1 interbody graft and iliac screws to support weak S1 screws and have significantly reduced the rates of pseudoarthrosis at the lumbosacral junction [18, 19]. The purposes of this study were to assess the comparative rates of pseudoarthrosis at the weaker points along the construct, the lumbosacral junction, and the level of osteotomy; estimate overall incidence of implant failure in long fusions for spine deformity; and comparatively analyze failures at different points along the construct.

## Methods

After Institutional Review Board (IRB) approval, a single center, retrospective analysis from the spine patient database was undertaken to identify patients who underwent surgical spinal deformity correction. Twenty-six patients were identified who had undergone tricolumnar wedge resection for correction of fixed spinal imbalance. All patients were managed by three surgeons from 2008 to 2011. Demographic data was recorded. Radiographs were made prospectively and reviewed retrospectively. Postoperative follow-up ranged from 12 to 54 months (mean 30 months).

The study population consisted of 26 patients (19 adults (12 female, 7 male; mean age 61.3 years (range 37 to 76 years)) and 7 pediatric patients). In the adult population, 16 were diagnosed with idiopathic scoliosis with flat back with or without previous spine surgery, one presented with de novo scoliosis with coronal imbalance, one diagnosed with charcot spine, and one with secondary thoracolumbar kyphosis related to a T12 burst fracture. Diagnoses for pediatric patients included congenital or early onset scoliosis in four and neuromuscular scoliosis in three patients.

Surgical data was collected from review of operative notes and intraoperative radiographs. Patients included in the study were grouped according to type of tricolumnar resection procedure: pedicle subtraction osteotomy (PSO, *n* = 18); vertebral column resection (VCR, *n* = 4); hemivertebra excision (HE, *n* = 2); or extracavitary corpectomy (EC, *n* = 2). The surgical technique common to all procedures included subperiosteal dissection of the spine at desired levels followed by securing fixation points proximal and distal to the planned level of osteotomy. Wide laminectomy was carried out at the level of osteotomy to allow inspection of the dura and neural elements and to avoid dural impingement when corrective forces were applied. Laminectomy was extended from a level of pedicles of the vertebra above to a level of pedicles of the vertebra below the level of resection. PSO was performed in patients with scoliosis and kyphoscoliosis with rigid structural curves in accordance with the technique described by Bridwell et al. [1]. Four patients demonstrating severe structural curves with higher than expected angle of correction or with truncal translation needed a VCR procedure performed utilizing a technique described by Suk et al. [12]. Holte et al. [20] described an effective procedure for correction of a congenital scoliotic curve resulting from asymmetric growth of the spine due to a hemivertebra which was utilized for correction in two children with segmented hemivertebrae. Two patients underwent corpectomy by an extracavitary approach in the thoracic region.

The vertebral level undergoing osteotomy and number of levels resected was noted for each patient. The number of levels included in the fusion, anchors used at most proximal and distal points of fixation, and percent saturation of fixation levels were recorded. Also, type of bone graft, bone graft substitutes, and use of bone morphogenic protein (BMP) were documented. Implant characteristics, specifically rod material and diameter, were noted whenever available.

Long cassette standing anteroposterior and lateral radiographs were made preoperatively and postoperatively at 2 weeks and 3, 6, and 12 months and annually thereafter. Radiographs were analyzed to determine anchor type used at most proximal and distal points of fixation, percent saturation of fixation levels and level of the osteotomy, C7 sagittal plumb line [21] (measured from center of C7 body to the posterosuperior corner of S1 body), and coronal plumb line (measured from center of C7 body to center of S1 body on an anteroposterior radiograph). Presence or absence of interbody structural graft at the lumbosacral junction was noted. Implant related to findings of screw loosening and/or fracture of the rod if the present level and laterality of the rod failure were recorded. A note was made if pseudoarthrosis was obvious on plain radiographs.

### Statistical methods

Failures were classified according to location either at osteotomy site or at a remote site. Continuous data was compared by paired *t* test. Chi-square analysis was used to compare demographic data of patients who had a failure of the hardware. Potential risk factors were also analyzed. *P* values <0.05 were considered significant.

## Results

The predominant type of corrective osteotomy in the adult population was PSO (18/19). The mean number of levels fused was 11.2 (range 2 to 16). Fusion was extended to the pelvis to include the lumbosacral junction in 17 adult patients, while L5 was the last instrumented vertebra in two adult patients due to healthy L5–S1 disc with minimal signs of degeneration. Iliac screws [22] were used in 16 adult patients to protect S1 screws in the relatively weak cancellous bone of the sacrum. Structural interbody graft was used in eight of the 17 patients with extension across lumbosacral junction. Transverse process hooks were used in eight patients as proximal most points of fixation, while pedicle screws were used in 18 patients supported by sublaminar wires in one patient with severely osteoporotic bone. A titanium rod was used to achieve and maintain correction in all adults. A stainless steel rod was utilized to hold correction in two children for better maintenance of correction due to the higher stiffness of the metal. Twenty-five patients had bilateral pedicle screws as fixation anchors two levels above and below the level of osteotomy. One patient with PSO at L3 level had only one screw at the adjacent L4 level as the other screw was removed due to fracture of the pedicle in osteoporotic bone during a compression maneuver. Autogenous bone graft obtained locally and from the iliac crest mixed with crushed cancellous allograft was used in all adults, and BMP was also utilized in seven adults who were either smokers or demonstrated pseudoarthrosis from previous surgery.

The level of the osteotomy was chosen based on a number of factors. The osteotomy was preferably done caudad to the level of conus medullaris wherever possible and at previously fused segments. Angular correction resulting from resection at one level was adequate for 22 patients, two patients needed more extensive osteotomy at two adjacent levels due to severe deformity, one patient needed unilateral decancellation of one adjacent segment in addition to the level of osteotomy to achieve coronal plane correction, and one patient underwent bone-disc-bone type of osteotomy to achieve optimal balance. Table 1 depicts the frequency of levels of osteotomy.

**Table 1:** Frequency of levels of osteotomy

Level of osteotomy	Number of patients
T8	1
T9	1
T10	1
T12	1
L1	3
L2	10
L3	3
L4	2
L5	2
L2 and L3	2

**Fig. 1:**
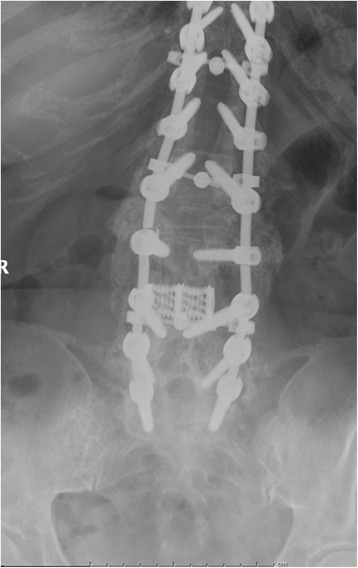
AP X-rays demonstrating fracture of rod at osteotomy site

**Fig. 2:**
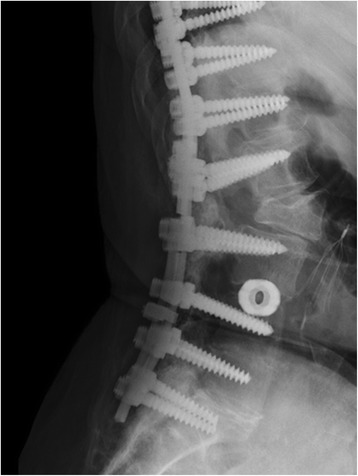
Lateral X-rays demonstrating fracture of rod at osteotomy site

**Fig. 3:**
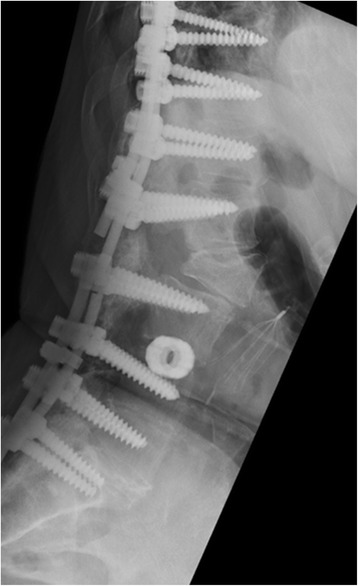
Flexion views demonstrate clear motion at the osteotomy

Thirty-seven percent of adults (7/19), i.e., 27% of patients overall (7/26 patients) demonstrated rod breakage either unilaterally or bilaterally during the follow-up. One failure (4%) was at the lumbosacral junction and six (23%) occurred at the osteotomy level having accompanying same level previous laminectomy (*P* = 0.014) (Figs. 1, 2, and 3). A broken rod was noted at the L2 level in three patients, L4 in two patients, and L3 in one patient (Table 2).

**Table 2:** Data of implant failure

Patient no.	Type of osteotomy	Level of osteotomy	Rod material	Level of rod failure
Patient 5	PSO	L1	Titanium	L5–S1
Patient 11	PSO	L2	Titanium	L2–L3
Patient 14	PSO	L4	Titanium	L4
Patient 19	PSO	L2	Titanium	L2
Patient 20	PSO	L2 and L3	Titanium	L2–L3
Patient 21	PSO	L4	Titanium	L4–L5
Patient 25	PSO	L2	Titanium	L2 on left, L3 on right

**Fig. 4:**
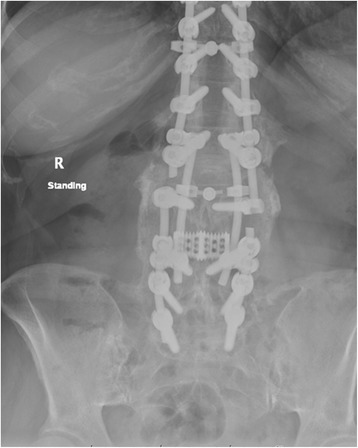
AP postoperative X-rays demonstrating rod repair

**Fig. 5:**
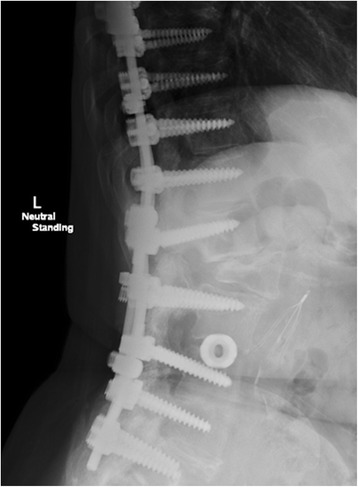
Lateral postoperative X-rays demonstrating rod repair

Failure of both rods was evident in five patients while two patients demonstrated a unilateral broken rod. A titanium rod was used in all patients with rod failures. Failure of the formation of bony trabeculae resulting in pseudoarthrosis was evident at the respective levels on plain radiographs. All patients who demonstrated implant failure at the level of osteotomy were between 6 and 12 months post surgery. Symptomatic rod failure occurred at 24 months in a patient with L5–S1 failure. There were no failures in patients with thoracic location of wedge resection and no failures in pediatric patients. Junctional kyphosis was apparent on follow-up radiographs in seven patients while only one patient was symptomatic with increased upper back pain. None developed any neurological symptoms as a result of worsening angulation due to a failure. This radiographic finding whenever evident was noted during midterm follow-ups frequently between 3 and 6 months after corrective surgery. Three patients with radiographic evidence of rod failure were symptomatic with increasing back pain, two experienced a steady decline in function due to worsening standing balance and back pain, and two with rod failure were completely asymptomatic. All symptomatic patients chose to have revision of failed implant addressed with exploration of pseuodoarthosis, replacement of the broken rod, and autogeneous iliac crest bone graft. BMP was used in three patients with revision after reviewing the benefits and possible adverse effects with these patients. A CT scan of the lumbar spine or flexion/extension views demonstrated solid arthrodesis in three of five patients (patients 5, 14, and 19) at 1 year follow-up. The other two patient revision surgeries did not demonstrate any evidence of implant failure at the latest visit; however, their follow-up was less than 1 year from surgery (Table 3) (Figs. 4 and 5).

**Table 3:** Patients with rod failures

Patient no.	Symptoms	Intervention	Outcome
Patient 5	Increasing back pain	Replacement of rod and use of lateral connectors Iliac crest bone graft	Fusion at the nonunion site
Patient 11	Increasing back pain	Rod replacement Use of BMP	Follow up less than 1 year No implant failure noted
Patient 14	Worsening posture Increasing back pain	Rod replacement Use of BMP	Fusion at the nonunion site
Patient 19	Worsening standing balance Back discomfort and muscle fatigue	Replacement of rod with lateral connectors Use of BMP	Fusion at the nonunion site
Patient 20	Asymptomatic	None	–
Patient 21	Increasing back pain	Rod replacement Iliac crest bone graft	Follow-up less than 1 year No implant failure noted
Patient 25	Asymptomatic	None	–

## Discussion

Tricolumnar osteotomy by posterior approach is a valuable tool in the spine surgeon’s armamentarium. PSO and VCR deal with more severe structural deformities with rigid curves where only posterior column osteotomy like Smith-Peterson or Ponte’s osteotomy may not be sufficient to achieve correction. Another advantage of these osteotomies is the ability to shorten the posterior column while achieving angular correction without lengthening of the anterior column, thus avoiding injury to major anterior visceral structures. Implant failures and pseudoarthrosis are common long-term complications associated with long fusions in spine deformities. The purposes of our study were to estimate the incidence of implant failures in deformity patients undergoing tricolumnar osteotomies and determine relative rates of failures at different points along the construct with analysis of possible factors leading to these failures.

Pseudoarthrosis rates have been reported to range from 17 to 24% in long fusions in a series by Kim et al. [23, 24]. In their analysis of 144 patients, 34 patients demonstrated pseudoarthrosis on follow-up radiographs. Almost all patients demonstrated this nonunion at junctional segments (17 at T10–L2 and 15 at L5–S1) in their study. High rates of implant failure have been reported at the osteotomy level in patients with PSO by Bridwell et al. and Yang et al. in their analysis of 33 and 35 patients, respectively [16, 25]. Our study demonstrated implant failure in 37% of adults and 27% of the overall study population. Six of seven (86%) of these failures were located at the level of osteotomy with accompanying previous laminectomies, whereas 14% (1/7) were located at the lumbosacral junction. This is comparable to 89% rod failure rate at the osteotomy site in patients undergoing PSO reported by Smith et al. in a multicenter retrospective review of 442 patients by the International Spine Study Group [26]. In our study, all patients had rod failures approximately 1 year from their surgery qualifying as “early” failures correlating with the International Spine Study Group’s findings. Interestingly, all of our failures of the osteotomy site occurred in PSO patients. Those patients were older as compared to the VCR and HE patients, and as such may have simply been more at risk patients with a higher potential for a non-union.

A comparatively low rate of rod breakage at the lumbosacral junction site seen in our study may be related to adopting the use of structural interbody graft and stronger iliac screws. This may have indirectly reflected a relatively higher rate at the previous laminectomy sites. Another caveat may be deficient posterior bone stock as a result of wide laminectomy essential to assess and prevent dural impingement during osteotomy closure. This is particularly relevant when the osteotomy is done through previously unfused segments and a surgeon must rely predominantly on anterior elements for arthrodesis. Complete closure of posterior elements or the base of the wedge by different maneuvers is ideal and desirable but may not always be practically possible, particularly in osteoporotic bone where balance needs to be achieved between adequate closure and preventing screw cutout or pullout. Optimal closure of the osteotomy wedge to achieve maximum bone surface contact as allowed by bone quality along with generous use of autograft to bridge the gap between two adjacent posterior elements is suggested. Efficacy of the bone morphogenic protein (RhBMP-2) and even its superiority over the iliac crest bone graft to enhance the rates of fusion has been well documented in long-term follow-up studies [27]. However, its use around the laminectomy site is limited in view of adverse effects over exposed neural elements and should be used judiciously. Finally, an apex of the new spinal curvature will be located at the osteotomy site, and maximum stress borne by the implants and maximum rod contouring at this point compounded by high notch sensitivity of titanium, especially during the early postoperative period until fusion occurs, and may be cited as one possible reason for relatively high implant failure rates at this location [28].

Another important finding was that 71% (5/7 patients) with an implant failure had increasing back pain or worsening sagittal balance or a combination of both, while two patients (29%) were asymptomatic and failure was an incidental finding on follow-up radiographs. Considering revision surgery is a significant endeavor for the patient both physically and mentally, a choice was offered to them to have it fixed surgically versus close observation. Being asymptomatic, both made an informed decision against surgical intervention but understood the possible need for revision surgery in the future if symptoms appear or function worsens.

The strength of our study is being one of the few studies attempting to analyze failures after three column osteotomy of the spine with direct comparison of failures at the osteotomy site and junctional levels. It highlights that maintenance of the correction, obtained through an osteotomy over time by optimizing rates of fusion through the segment, is as crucial to the success of deformity surgery as achieving this correction with intraoperative maneuvers. The primary limitation of our study is its retrospective nature. Also, longer follow-ups may increase the number of rod failures recorded. A prospective study with randomization of patients is warranted but may not be feasible due to ethical issues and expected high rates of crossover based on intraoperative decisions. Nonetheless, this study was an attempt to directly compare rates of implant failure at different points along the construct and highlight possible factors which may be related to differences in the rates of failure.

## Conclusion

The overall incidence of rod/implant failure noted after three column osteotomies for deformity correction was 27%. Among these, 86% occurred at the level of osteotomy as compared to 14% at the junctional level. Possible reasons for this significant difference are increased stress in the rod at this point as a result of maximum contouring and relatively deficient posterior bone stock secondary to wide laminectomy. The observation is also likely a result of improved fusion rates at the lumbosacral junction consequent to adoption of interbody structural graft and strong iliac screws. However, additional biomechanical studies are needed to assess the importance of these factors.

## Abbreviations

BMP: Bone morphogenic protein
EC: Extracavitary corpectomy
HE: Hemivertebra excision
IRB: Institutional Review Board
PSO: Pedicle subtraction osteotomy
VCR: Vertebral column resection
